# Fabrication of a Flexible, Wireless Micro-Heater on Elastomer for Wearable Gas Sensor Applications

**DOI:** 10.3390/polym14081557

**Published:** 2022-04-11

**Authors:** Jonam Cho, Gunchul Shin

**Affiliations:** School of Materials Science and Engineering, University of Ulsan, 12 Technosaneop-ro 55 beon-gil, Nam-gu, Ulsan 44776, Korea; dyfska@naver.com

**Keywords:** micro-heater, wireless, NFC, PDMS, gas sensor, wearable, SnO_2_ nanowire, flexible

## Abstract

Thin-film microdevices can be applied to various wearable devices due to their high flexibility compared to conventional bulk-type electronic devices. Among the various microdevice types, many IoT-based sensor devices have been developed recently. In the case of such sensor elements, it is important to control the surrounding environment to optimize the sensing characteristics. Among these environmental factors, temperature often has a great influence. There are cases where temperature significantly affects the sensor characteristics, as is the case for gas sensors. For this purpose, the development of thin-film-type micro-heaters is important. For this study, a wirelessly driven thin-film micro-heater was fabricated on the flexible and stretchable elastomer, a polydimethylsiloxane (PDMS); the antenna was optimized; and the heater was driven at the temperature up to 102 degrees Celsius. The effect of its use on gas-sensing characteristics was compared through the application of the proposed micro-heater to a gas sensor. The heated SnO_2_ nanowire gas sensor improved the performance of detecting carbon monoxide (CO) by more than 20%, and the recovery time was reduced to less than half. It is expected that thin-film-type micro-heaters that can be operated wirelessly are suitable for application in various wearable devices, including those for smart sensors and health monitoring.

## 1. Introduction

Smart wearable devices for biomedical applications and healthcare, which are attached to the outside of the skin or inserted into the body to monitor various data, such as pressure, temperature, humidity, UV index, oxygen saturation, pulse, and sweat, have been the subject of many studies recently [1,2,3,4,5,6,7,8,9,10,11]. Additionally, they can be used to deliver drugs, heat, light, and so on for various therapeutic purposes [12,13,14]. There have been many recent reports of fatal accidents caused by gases that are harmful to the human body. As existing gas sensors are bulky and not easy to carry, new sensor platforms with maximized portability and functionality, such as micro-gas sensors and smart gas sensors, are required. Unlike existing silicon-based electronics, devices that are flexible or wearable applicable should be manufactured using plastic-based flexible materials. However, since the existing semiconductor process includes high-temperature thin-film deposition and patterning processes, soft polymer-based flexible electronics with a low processing temperature have limitations. Therefore, polymer materials such as polyimides, which are stable even at a high temperature, and silicone polymer, which is one of elastomers, were used to ensure stretchable characteristics beyond the flexible characteristics of materials normally used [15,16,17]. Among them, PDMS has been used in various wearable and body implantable devices due to its low modulus, high flexibility, optical transparency, chemical stability, high processability, and biocompatible properties [5,6,7,8,9,10,13,17]. In addition, it showed stable characteristics even at temperatures of 100 degrees Celsius or higher, making it possible to use it as a substrate material for a micro-heater in this study. The heating devices are used to increase the temperature of the gas sensor, allowing it to measure the desired gas with high sensitivity and specificity [18,19,20,21,22,23,24,25]. Unlike existing gas sensors, a thin-film micro-heater is required for application in a portable or wearable gas sensor. Thin-film battery or wireless power transmission technology can be used to drive the micro-heater, but its application is limited due to high power consumption [19,26]. In this study, we describe the fabrication of a wireless micro-heater coated with Pt, which can heat up to 124 degrees Celsius and can receive a near-field communication (NFC)-based 13.56 MHz radio frequency signal using an antenna coil made of thin aluminum. On changing the geometry of the receiving antenna and the micro-heater, changes in the performance of the wireless heater were confirmed and sensing performance improvement was achieved when applying a tin oxide (SnO_2_) nanowire element to a carbon monoxide (CO) gas sensor. In the case of a nanowire gas sensor that detects gas due to a change in the current flowing in a semiconductor nanowire, the optimal sensing temperature is divided depending on the type of gas, and this research compares the sensing characteristics while heating SnO_2_ nanowire gas sensor device at 100 degrees Celsius or higher [7,20,27]. The target was about 100 degrees Celsius as a temperature condition for optimizing the stable temperature and the sensing capability of the PDMS used in this study. In particular, in the case of SnO_2_ nanowires, the adsorption of various gases, especially water molecules, has a significant effect on the change in current characteristics and on the change in the recovery time caused by the desorption of water molecules at a temperature of 100 degrees Celsius or higher.

## 2. Materials and Methods

**Figure 1:**
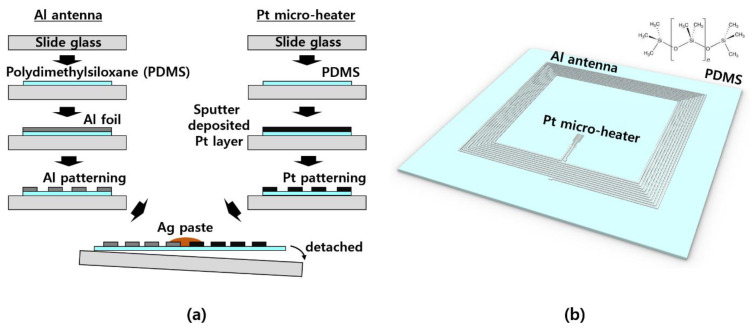
Schematic illustration of the fabrication procedure (**a**) and perspective view (**b**) of a wireless micro-heater device.

**Figure A1:**
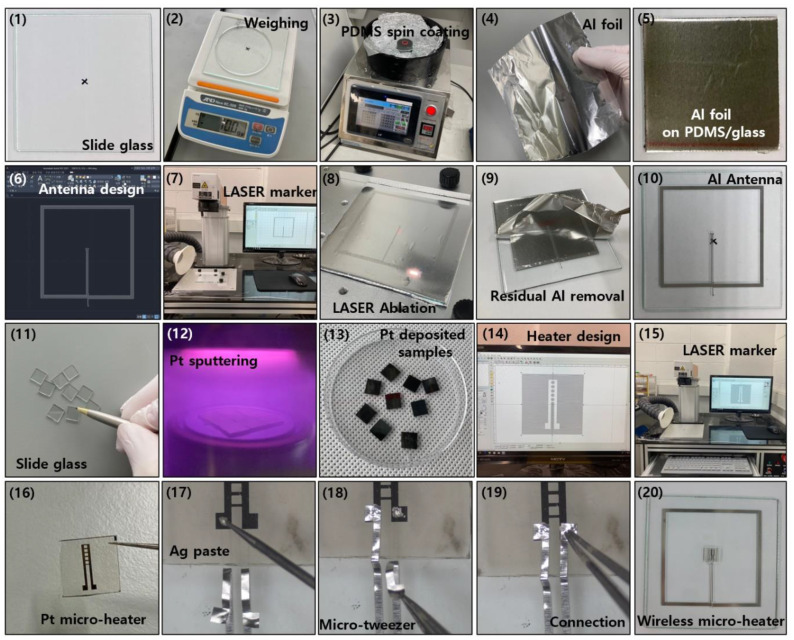
Photographs of the detailed fabrication procedure of a wireless micro-heater consisting of an Al antenna and a Pt micro-heater.

The fabrication of the wireless micro-heater was completed by manufacturing and connecting two elements: an aluminum antenna and a micro-heater. The fabrication process is shown in Figure 1a. We started with the production of polydimethylsiloxane (PDMS), which is the most-used polymer material among elastomers. PDMS was produced by mixing Sylgard 184 (Dow Corning, Midland, MI, USA) and a curing agent in a mass ratio of 10:1 and was crosslinked at room temperature for more than 48 h to remove air bubbles [7,13,28,29,30]. In the case of the aluminum antenna, aluminum foil was attached to a PDMS piece placed on a glass slide and LASER ablation was performed to create the antenna pattern using LASER marker equipment (Hyosung Laser, Bucheon, Korea). The micro-heater was coated with Pt to a thickness of 100 nm, using sputter deposition equipment (Ion sputter coater, G20, GSEM, Suwon, Korea) on the PDMS piece attached to the slide, and then a fine pattern was produced through LASER ablation in the same way. Finally, the antenna and the micro-heater were electrically connected using Ag paste (Silver conductive epoxy 8330, MG chemicals, Surrey, BC, Canada) and then removed from the slide to complete the device. The detailed fabrication method is shown in Figure A1. Without general microfabrication equipment, device fabrication can be completed within 4 h using simple equipment, such as a spin coater (SP-6, Prowin, Daejeon, Korea), a LASER marker, a stereomicroscope (Stereomicroscope, SDPTOP, Optika, Via Rigla, Italy), and desk-type sputter equipment. Detailed processes can be found in previous reports, as well [7,28,29].

## 3. Results and Discussion

The completed wireless micro-heater can be seen in Figure 1b. The wireless micro-heater element, composed of an Al antenna and a Pt heater, was manufactured on a PDMS substrate and had a minimum thickness of 50 μm, depending on the process. It being in the form of a flexible polymer thin-film, it is expected that wearable applications will be possible.

### 3.1. Wireless Micro-Heater and the Power Transfer System

**Figure 2:**
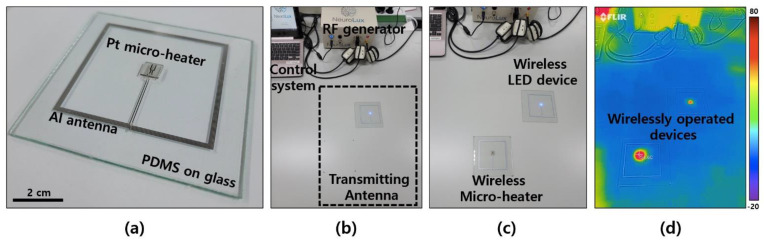
Wireless micro–heater and NFC–based power transfer system. (**a**) Wireless micro-heater device on a PDMS/glass substrate. (**b**) The wireless power transfer system consists of a RF generator, a control system, and a transmitting antenna. In (**c**,**d**) are a photograph and an IR image of wirelessly operated LED and micro-heater devices, respectively.

A photo of the fabricated sample is shown in Figure 2a. The device in the photo was placed on a glass slide for handling, and the micro-heater device connected to an Al antenna with an outer diameter of 74 mm × 74 mm is shown. The operation of the heater element is driven by an external NFC-based wireless power transmission device (Neurolux, Chicago, IL, USA). Figure 2b shows a wireless system that can create and control a 13.56 MHz radio signal. An NFC-based wireless power transfer system, Neurolux in here, offered diverse expandability on the experimental apparatuses and environment compared with the wireless system using higher radio frequencies, such as a surrounding conductive material [30,31]. This wireless transmission system consists of a radio frequency (RF) generator, a control system, and a transmitting antenna. The transmitting antenna was installed under the experimental table in the area shown in Figure 2b and was set up to enable wireless power transmission up to 20 cm in the height direction through the antenna installed, based on an area of approximately 40 cm × 50 cm. Figure 2c and 2d show photos of the micro-heater being wirelessly driven by the power transmission system and a temperature gradient image obtained with an IR camera (FLIRONE PRO, FLIR, Wilsonville, OR, USA), respectively. It can be driven with a transmit power of up to 10 W from a transmit antenna installed 3 cm below the table, and the IR image in Figure 2d shows that the wireless light-emitting diode (LED) device and the wireless micro-heater were successfully driven using the corresponding wireless power transmitter. A wireless LED element was also manufactured with the same receiving antenna, which was placed together with the micro-heater to confirm that the wireless operation was performed successfully.

### 3.2. Antenna Performance

**Figure 3:**
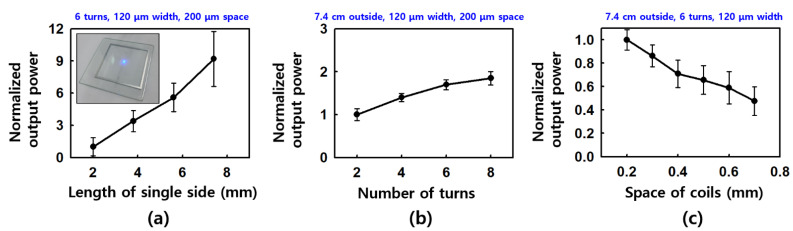
Antenna performance comparison of the receiving antennas according to the length of the outer side (**a**), the number of rotations (**b**), and the spacing of the coil (**c**), respectively. The inset image of (**a**) shows the wirelessly operated LED device with a connected antenna.

To analyze the performance of the wireless driving antenna and to easily check the optimal antenna shape, the output power performance of the LED driven by the power received by the antenna was compared by connecting an LED [31] instead of a micro-heater. The inset image in Figure 3a shows the operation of the LED with a connected wireless antenna. The performance of the receiving antenna is determined by the outer diameter of the antenna coil, the number of turns of the coil, the spacing between coils, the resistance of the coil material, the width of the coil, and the inner diameter of the coil. In our experiment, we considered the variables having the greatest influence to be the outer diameter, the number of rotations, and the spacing. The coil’s resistance and width have little effect when using a thick aluminum material, which has a low specific resistance (~2.65 Ω·m at room temperature). Furthermore, in the case of the inner diameter, it is a variable that is automatically determined by outer diameter, number of rotations, width, and spacing. Figure 3a, 3b, and 3c show the results of a performance comparison of the receiving antennas according to the length of the outer side, the number of rotations, and the spacing of the coil, respectively. Under the same transmitting antenna driving conditions, other variables were equally controlled and the performance of the receiving antenna was compared based on the change in LED brightness. In the case of the length of the outer side, it was confirmed that the receiving performance was improved as the length of the outer side of the square-shaped antenna increased. The length was compared by increasing it from 2 cm to 7.4 cm (in 1.8 cm steps), and it was confirmed that the performance increased as the length of one side increased. This was due to the larger antennas showing the characteristics of a receiving antenna, having a peak frequency closer to 13.56 MHz, without proper impedance matching using a capacitor between the coil lines. In addition, it appears that the receiving performance improved as the number of rotations of the antenna coil increased; however, unlike the result when considering the outer diameter, there was no dramatic improvement. This is thought to be because the inner diameter decreases as the number of rotations increases, as well as because the overall length of the coil becomes longer and the resistance increases (R ∝ L). It is expected that the performance can be further improved by introducing a multi-stacked antenna type, which increases the number of rotations while maintaining the size of the inner diameter. It was found that the smaller the spacing between the coils, the better the reception performance, and the characteristic of the experimental equipment showed a steady improvement in performance up to about 200 μm, which is the diameter of the LASER (80 μm) plus the minimum center spacing. Accordingly, to optimize the performance of the receiving antenna, we determined that it is advantageous to increase the size of the outer diameter, increase the number of turns within the range where the inner diameter does not decrease, and keep the spacing between the coils to a minimum.

### 3.3. Performances of Wireless Micro-Heaters

**Figure 4:**
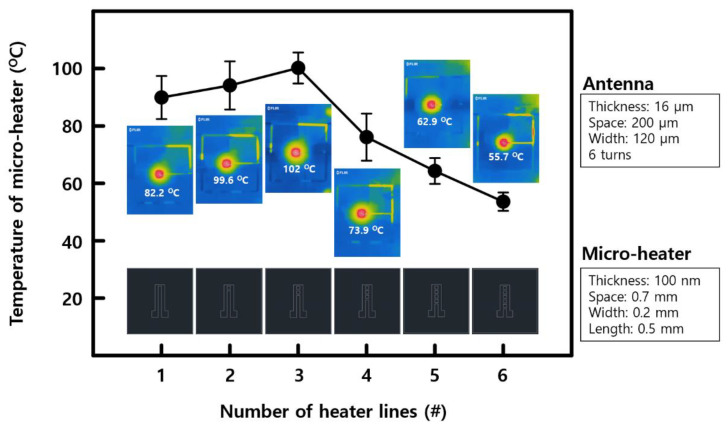
Average temperature of the heater elements as a function of the number of heater lines with the transmitting power of 10 W. The temperature was 102 degrees Celsius, the highest temperature in three heater lines, and about 55 degrees Celsius, the lowest temperature in six heater lines.

**Figure A2:**
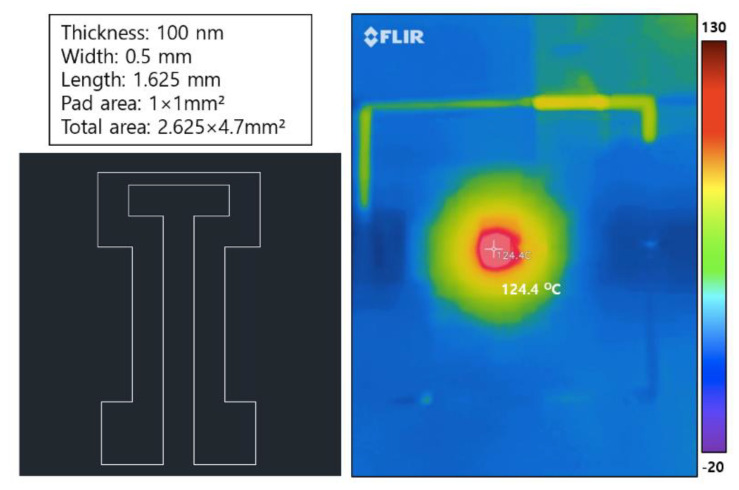
Scheme of a micro-heater. The IR image shows the heating temperature up to 124.4 degrees Celsius.

Based on the above results, the heating performance when operating wirelessly using an antenna with a side length of 7.4 cm, 6 turns, and a line spacing of 0.2 mm connected to a micro-heater with 1–6 heating lines was confirmed using an IR camera. As shown in Figure 4, the average temperature of the heater elements was measured by changing the number of heating lines after setting the transmission power to 10 W, the heating line spacing of the micro-heater to 0.7 mm, the width to 0.2 mm, and the length to 0.5 mm. The time required to reach the measured temperature was fixed at 1 min in all cases. In the figure, the IR image results for the heaters are shown above, while the schematic of each heater is shown below. A heating temperature of about 102 degrees Celsius was reached with the given antenna and heater geometry, that is, using a single-layer antenna without proper impedance matching. It is expected that a higher heater temperature can be obtained by using a multi-stack configuration for the antenna or by configuring the geometry of the heater differently. As shown in Figure A2, we confirmed that heating of up to 124 degrees Celsius can be achieved even when using a heater with only one heating line. As the geometry of the antenna greatly affects the performance, there are various variables that may be considered, such as thickness, length, width, number of lines, and line shape, in the case of micro-heaters.

### 3.4. Wireless Micro-Heater for a Gas-Sensing Application

**Figure 5:**
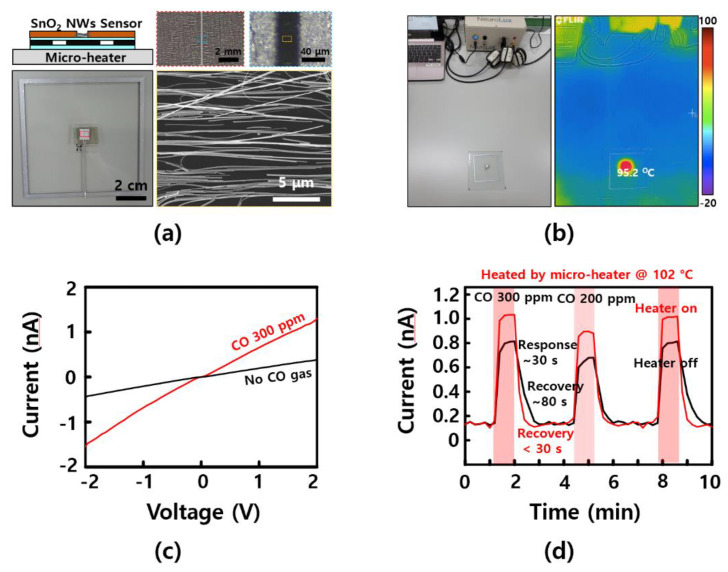
Micro-heater for CO–gas–sensing applications. (**a**) Images of a SnO_2_ nanowire gas sensor device; (**b**) photograph and IR image of a wirelessly operated micro-heater with a SnO_2_ nanowire gas sensor; (**c**) current–voltage (IV) curve of a SnO_2_ nanowire device without CO gas (black line) and with 300 ppm CO gas (red line); and (**d**) current on the SnO_2_ nanowire as a function of the CO gas level without a heater (black line) and with a heater (red line).

**Figure A3:**
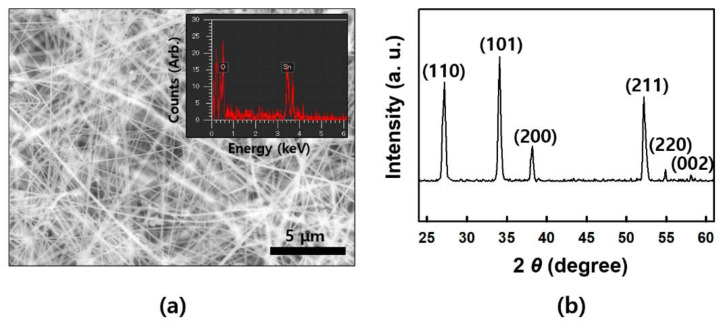
As-grown SnO_2_ nanowires. (**a**) Scanning electron microscopy (SEM) image and energy-dispersive X-ray spectroscopy data (inset). (**b**) X-ray diffraction (XRD) graph of rutile SnO_2_ nanowires.

Figure 5a shows a SnO_2_ nanowire gas sensor fabricated by patterning aluminum electrodes after aligning and transferring SnO_2_ nanowires grown by CVD using sliding transfer technology [7,32,33]. Figure A3 shows an SEM image of as-grown nanowires, elemental analysis, and crystallinity characteristics of the SnO_2_ nanowire used in this study. When the nanowires grown vertically on the substrate are transferred to the opposite substrate, by applying a constant pressure and speed to the substrate, it can be confirmed that the nanowires were aligned in the sliding direction, as shown in Figure 5a. A SnO_2_ nanowire device having a channel length of 40 μm was fabricated after sliding transfer onto a thin PDMS (10 μm thickness), on the previously fabricated wireless micro-heater device. Using the wireless power transmission system, whether the wireless micro-heater on which the SnO_2_ nanowire gas sensor was mounted operated wirelessly was monitored using an IR camera (Figure 5b). SnO_2_ nanowires are widely used for applications involving sensing various gaseous materials, including carbon monoxide (CO), due to their high surface/volume ratio and changes in electrical properties according to oxygen vacancy [7,27,34]. However, the temperature at which the sensing characteristic is maximal differs for various gases and, in the case of CO, it has been reported that the sensing power is increased at about 100 degrees Celsius or higher [20,27]. In addition, as the characteristics are affected by the surrounding humidity, it is known that a temperature of 100 degrees Celsius or higher, which can remove water molecules, can improve the sensor performance (e.g., reducing the recovery time). Therefore, using the wireless micro-heater manufactured in this study, the change in CO gas detection performance was confirmed when heating to 102 degrees Celsius, comparing the results obtained when using the heater and when it was not used. First, the change in current, according to the carbon-monoxide-gas-sensing ability of the prepared SnO_2_ nanowire device, was confirmed. In Figure 5c, the current difference was about 10 times the source–drain voltage difference of 2 V in the presence of 300 ppm of carbon monoxide gas. As shown in Figure 5d, the current through the SnO_2_ nanowire gas sensor element differed by more than 20% when the heater was driven compared to when it was not. Water molecules could be easily removed, allowing for shorter recovery time than when no heater was used. This result is similar to or higher than the previously reported CO gas sensor sensitivity of the resistive type and lower than the transistor type with an amplified result. In addition, the recovery time results show higher recovery speed results compared to previously reported results [7,19,20,23,25]. If a higher heating temperature were obtained—for example, through additional geometric modification of the antenna and the heater—we expect that better sensing characteristics for various gases can be obtained, with optimal sensitivity at higher temperatures [35,36].

### 3.5. Flexible Properties of a Wireless Micro-Heater

**Figure 6:**
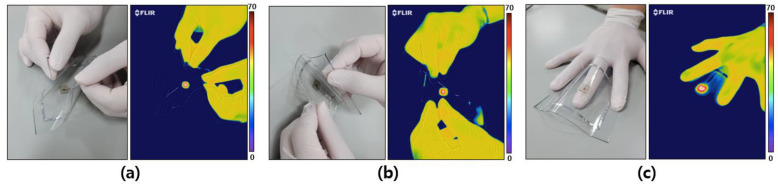
Wireless operation of a micro-heater with various modifications, such as bending in the diagonal direction (**a**), the horizontal direction (**b**), and as if putting it between fingers (**c**).

For wearable gas sensor applications, it is necessary to implement the device in a soft and flexible form as well as with gas-sensing characteristics and wireless driving function. Unlike the conventional device platforms, to apply the device to our skin or clothes, the device must be made of a material having a low modulus. This is because conventional semiconductor-based devices have a high modulus (~GPa), so a problem may occur due to the modulus mismatch with a surface with a low modulus (~kPa), such as skin. In this study, the micro-heater and the wireless antenna were fabricated on PDMS with a low modulus, and when finally removed from the glass substrate, the micro-heater had a flexible form, as shown in Figure 6. The antenna was subjected to various modifications, such as bending in the diagonal direction, bending in the horizontal direction, and as if putting it between fingers, and at the same time, the micro-heater was driven wirelessly. Through the related photos and infrared images in Figure 6a–c, it can be confirmed that the heater element is operated wirelessly despite deformation, such as bending. It is expected that the wirelessly operated micro-heater manufactured on the above soft material platform can be applied directly to the skin or clothing to be used in various wearable applications and sensors.

The results of the micro-heaters and gas sensors produced in this study are shown in a table together with other previously reported devices. Table 1 summarizes the maximum heating temperature of the heater, sensor type, sensor material, sensing purpose, sensitivity, recovery time, flexibility, substrate material, and the wireless availability of devices composed of various gas sensors and micro-heaters.

## 4. Conclusions

A wireless micro-heater device was fabricated through simple patterning of Al and Pt thin-films onto a flexible polymer-based material. The performance of the micro-heater was improved by modifying the geometry of both the receiving antenna and the micro-heater. The antenna performance showed a difference of up to 9 times as the length of one side increased, up to around 1.8 times as the number of turns in the coil increased, and up to 2 times as the coil space increased, so the micro-heater temperature was driven from 55 degrees Celsius to 102 degrees Celsius. It was also confirmed that the CO-sensing capacity of the SnO_2_ gas sensor was improved by up to 20% at 102 degrees Celsius based on 300 ppm of CO gas. In addition to the results obtained in this study, it is expected that a more efficient wireless micro-heater system can be made by conducting additional research, for example, by improving the geometry of both parts of the device, the antenna and the micro-heater. The developed wirelessly operable micro-heater can be used in various wearable and healthcare devices that require temperature control, which can improve the associated sensing performance, such as that in gas sensors. In the future, high usability is expected to be facilitated through integration with various wireless systems, such as IoT-based smart sensors and healthcare monitoring systems.

## Figures and Tables

**Table 1:** Various reported devices consisting of gas sensors and micro-heaters.

Properties	This Work	Chang et al., 2002 [37]	Kang et al., 2017 [20]	Hwang et al., 2011 [19]	Yeh et al., 2020 [25]	Lee et al., 2021 [23]	Shin et al., 2020 [7]
Device	Heater and sensor	Heater and sensor	Heater and sensor	Heater and sensor	Heater	Heater and sensor	Sensor
Max. heating temperature	102 °C	500 °C	210 °C	460 °C	45 °C	420 °C	-
Sensor type	Resistive	Resistive	Resistive	Non dispersive IR	-	Resistive	Transistor
Sensing material	SnO_2_ nanowire (NW)	Al-doped ZnO film	SiNx membrane	SiNx	-	Carbon nanotube	SnO_2_ nanowire (NW)
Purpose	CO sensing	CO sensing	CO sensing	Gas sensing	Hyperthermia treatment	Gas sensing	Humidity alarm
Sensitivity ^1^	~10	~2	25 ppm	-	-	~1.1	~10^4^
Recovery	~30 s	~130 s	-	-	-	~39 s	Slow
Flexibility	Flexible	Rigid	Rigid	Rigid	Flexible	Rigid	Flexible
Substrate	PDMS	Silicon	Silicon	Silicon	PDMS	Silicon	PDMS
Wireless availability	NFC-based wireless	-	-	-	Wireless	-	NFC-based wireless

^1^ Resistance (or current) change depending on the presence of gas or the minimum sensing concentration.

## Data Availability

Not applicable.

## Appendix A

Figure A1 shows the detailed procedure for fabricating the wireless micro-heater that is described here. The heating performance and related images of the additional optimized geometry of the micro-heater are shown in Figure A2. Figure A3 represents the scanning electron microscope (SEM) image, energy-dispersive X-ray spectroscopy data, and X-ray diffraction data of the SnO_2_ nanowires used here.
